# A novel immunoscintigraphy technique using metabolizable linker with angiotensin II treatment

**DOI:** 10.1038/sj.bjc.6990286

**Published:** 1999-04

**Authors:** Y Nakamoto, H Sakahara, T Saga, N Sato, S Zhao, Y Arano, Y Fujioka, H Saji, J Konishi

**Affiliations:** Department of Nuclear Medicine and Diagnostic Imaging, Graduate School of Medicine, Kyoto University Hospital, 54 Shogoin-kawahara-cho, Sakyo-Ku, Kyoto, 606-8507, Japan; Department of Patho-Functional Bioanalysis, Graduate School of Pharmaceutical Science, Kyoto University, Kyoto, Japan

**Keywords:** monoclonal antibody, tumour imaging, metabolizable linker, angiotensin, induced hypertension

## Abstract

Immunoscintigraphy is a tumour imaging technique that can have specificity, but high background radioactivity makes it difficult to obtain tumour imaging soon after the injection of radioconjugate. The aim of this study is to see whether clear tumour images can be obtained soon after injection of a radiolabelled reagent using a new linker with antibody fragments (Fab), in conditions of induced hypertension in mice. Fab fragments of a murine monoclonal antibody against human osteosarcoma were labelled with radioiodinated 3′-iodohippuryl N-ɛ-maleoyl-L-lysine (HML) and were injected intravenously to tumour-bearing mice. Angiotensin II was administered for 4 h before and for 1 h after the injection of radiolabelled Fab. Kidney uptake of ^125^I-labelled-HML-Fab was much lower than that of ^125^I-labelled-Fab radioiodinated by the chloramine-T method, and the radioactivity of tumour was increased approximately two-fold by angiotensin II treatment at 3 h after injection, indicating high tumour-to-normal tissue ratios. A clear tumour image was obtained with ^131^I-labelled-HML-Fab at 3 h post-injection. The use of HML as a radiolabelling reagent, combined with angiotensin II treatment, efficiently improved tumour targeting and enabled the imaging of tumours. These results suggest the feasibility of PET scan using antibody fragment labelled with ^18^F-fluorine substitute for radioiodine. © 1999 Cancer Research Campaign


*British Journal of Cancer* (1999) **79**(11/12), 1794–1799
© 1999 Cancer Research Campaign
Article no. bjoc.1998.0286

### A novel immunoscintigraphy technique using metabolizable linker with angiotensin II treatment

**Y Nakamoto[1], H Sakahara[1], T Saga[1], N Sato[1], S Zhao[1], Y Arano[2], Y Fujioka[2], H Saji[2] and J Konishi[1]**

[1]Department of Nuclear Medicine and Diagnostic Imaging, Graduate School of Medicine, Kyoto University Hospital, 54 Shogoin-kawahara-cho, Sakyo-Ku, Kyoto 606-8507, Japan; [2]Department of Patho-Functional Bioanalysis, Graduate School of Pharmaceutical Science, Kyoto University, Kyoto, Japan

**Summary** Immunoscintigraphy is a tumour imaging technique that can have specificity, but high background radioactivity makes it difficult to obtain tumour imaging soon after the injection of radioconjugate. The aim of this study is to see whether clear tumour images can be obtained soon after injection of a radiolabelled reagent using a new linker with antibody fragments (Fab), in conditions of induced hypertension in mice. Fab fragments of a murine monoclonal antibody against human osteosarcoma were labelled with radioiodinated 3′-iodohippuryl N-ε-maleoyl-L-lysine (HML) and were injected intravenously to tumour-bearing mice. Angiotensin II was administered for 4 h before and for 1 h after the injection of radiolabelled Fab. Kidney uptake of $^{125}$I-labelled-HML-Fab was much lower than that of $^{125}$I-labelled-Fab radioiodinated by the chloramine-T method, and the radioactivity of tumour was increased approximately two-fold by angiotensin II treatment at 3 h after injection, indicating high tumour-to-normal tissue ratios. A clear tumour image was obtained with $^{131}$I-labelled-HML-Fab at 3 h post-injection. The use of HML as a radiolabelling reagent, combined with angiotensin II treatment, efficiently improved tumour targeting and enabled the imaging of tumours. These results suggest the feasibility of PET scan using antibody fragment labelled with $^{18}$F-fluorine substitute for radioiodine.

**Keywords:** monoclonal antibody; tumour imaging; metabolizable linker; angiotensin; induced hypertension

Immunoscintigraphy using a monoclonal antibody is a technique that makes it possible to get a specific image of a tumour (Corbisiero et al, 1991; Collier et al, 1992; Gasparini et al, 1994). However, delays caused by slow clearance of radioactivity from normal organs prevent it from obtaining images soon after the administration of radiolabelled reagent. Use of antibody fragments (Fab) accelerates blood clearance of the radiolabel, but the renal uptake of radiolabelled fragments is higher than that of intact antibodies and the absolute amount accumulated in tumours is relatively low.

Recently, we developed a new metabolizable linker, designated HML, designed so that radioiodinated HML-protein conjugate is metabolized and rapidly cleared from the body (Wakisaka et al, 1997). The radioiodinated HML-protein conjugate is stable in the serum but is rapidly metabolized in the liver to release m-iodohippuric acid, which is cleared through the kidneys.

Vasoactive agents, such as angiotensin II, by producing vasoconstriction in normal tissue, are known to divert arterial blood selectively toward tumours and thereby enhance the delivery of drug-loaded particles (Suzuki et al, 1981; Hori et al, 1985; Hori et al, 1993). Vasoactive agents have already been used clinically to obtain high accumulations of systemically administered chemotherapeutic drugs to malignant tumours, such as gastric carcinoma (Sato et al, 1995). By combining the use of antibody fragments radioiodinated with HML and angiotensin II treatment, it may be possible to obtain high tumour uptake of radiolabelled antibody with low background radioactivity sooner, which would make it possible to obtain a clear image of the tumour soon after administration of radiolabelled reagent. In the present study, we investigated the effect of HML-radioiodination and angiotensin II treatment on tumour uptake of radiolabelled antibody fragment in tumour-bearing mice, and whether clear tumour images could be obtained soon after administration of radiolabelled antibody fragment.

*Received 5 June 1998*
*Revised 17 August 1998*
*Accepted 13 October 1998*

*Correspondence to:* Y Nakamoto

### MATERIALS AND METHODS

### Experimental tumour

KT005 human osteosarcoma cells (Sakahara et al, 1987) were grown in RPMI 1640 (Nissui, Tokyo, Japan) containing 10% fetal calf serum (GIBCO Laboratories, Grand Island, NY, USA) and 0.03% L-glutamine at 37°C in 5% $CO_2$. Subconfluent cells were removed from the culture dishes using calcium- and magnesium-free phosphate-buffered saline (PBS) containing 0.02% EDTA to preserve antigenicity, and $5 \times 10^6$ KT005 cells were inoculated subcutaneously into female Balb/c nu/nu mice. Tumours grew to approximately 800 mg 3 weeks after the inoculation. The tumours were maintained by serial passage of subcutaneous tumour in the nude mice.

### Monoclonal antibodies and preparation of Fab fragments

The OST7 antibody ($IgG_1$ isotype) was raised using human osteogenic sarcoma (Hosoi et al, 1982), and it has been shown to react with human osteogenic sarcoma cells with an alkaline phosphatase-related substance as an antigen (Tanaka et al, 1986; Nakamura et al, 1987). OST7 antibody was purified from the ascites of hybridoma-bearing mice using Protein A column chromatography (Bio-Rad, Richmond, CA, USA). Monoclonal antibody 56C ($IgG_1$), which recognizes human chorionic gonadotropin, was used as the isotype-matched control antibody (Kobayashi et al, 1993). Fab fragments were generated by papain digestion of the whole IgG of OST7 and 56C. Papain was added to IgG in 0.075 M phosphate buffered saline, pH 7.0, to yield an enzyme–IgG weight ratio of 1:33. After incubation at 37°C for 1 h, the reaction was stopped by adding 10% volume of 0.5 M iodoacetamide. The Fab fragments were separated by Superdex 200 column chromatography (Pharmacia Biotech, Uppsala, Sweden) and were further purified from contaminated Fc fragments by Protein A affinity chromatography (Bio-Rad, Richmond, CA, USA).

### Radioiodination of Fab

Radioiodination of Fab fragments was done by a two-step procedure (Arano et al, 1994; Wakisaka et al, 1997). First, HML was radioiodinated, and the radioiodinated HML was then conjugated to the Fab fragment. HML was dissolved in methanol containing 1% acetic acid (0.64 mg $ml^{-1}$), and 32.6 µl of this solution was mixed with 4 µl (14.8 MBq; 400 µCi) of Na[$^{125}$I] (DuPont, Wilmington, DE, USA). After addition of 8.88 µl of *N*-chlorosuccinimide (NCS) in methanol (0.5 mg $ml^{-1}$), the reaction mixture was incubated at room temperature for 25 min. Aqueous sodium bisulphite (4.44 µl, 0.72 mg $ml^{-1}$) was then added to quench the reaction. The radiochemical yield of [$^{125}$I]-HML was determined by TLC developed with a mixture of chloroform–methanol–acetic acid (40:5:2). Methanol was removed by an $N_2$ flow prior to subsequent conjugation reaction with Fab.

Before conjugation with [$^{125}$I]-HML, Fab was treated with 2-iminothiolan (IT), as previously reported (Arano et al, 1994). Briefly, 14.4 µl of freshly prepared 2-IT (1.0 mg $ml^{-1}$) in well-degassed 0.1 M borate buffer (BB; pH 8.0) containing 2 mM EDTA was added to 250 µl of Fab (2 mg $ml^{-1}$) in the same buffer. After gentle stirring at room temperature for 1 h, excess 2-IT was removed by the centrifuged column procedure (Meares et al, 1984) using a Sephadex G-50 gel (Pharmacia Biotech, Uppsala, Sweden) equilibrated with well-degassed 0.1 M PB (pH 6.0) containing 2 mM EDTA. The filtrate solution (200 µl) was then added to the reaction vial containing 12.6 MBq of crude [$^{125}$I]-HML. After gentle agitation of the reaction mixture for 1.5 h at room temperature, 29.6 µl of iodoacetamide (10 mg $ml^{-1}$) in 0.1 M PB (pH 6.0) was added into the mixture and incubated for 30 min. Then radiolabelled Fab was purified by the centrifuged column procedure using a Sephadex G-50 gel equilibrated with 0.1 M PB (pH 7.4). The specific activities of the $^{125}$I-HML-IT-Fab (OST7) and $^{125}$I-HML-IT-Fab (56C) were about 0.7 MBq $mg^{-1}$ and 0.5 MBq $mg^{-1}$, respectively. Fab fragments were also radioiodinated by the chloramine-T method (Hunter et al, 1962; Greenwood et al, 1963). Purified Fab (50 µg) of OST7 in 0.3 M phosphate buffer (pH 7.5) and $^{125}$I (11.1 MBq) were mixed with 2.5 µg of chloramine-T (Nakarai Tesq, Kyoto, Japan) dissolved in 0.3 M phosphate buffer. After reacting for 5 min, the radiolabelled Fab was separated from free iodine by PD-10 gel chromatography (Pharmacia, Uppsala, Sweden). The specific activity of the $^{125}$I-labelled-Fab was about 60.8 MBq $mg^{-1}$.

### Cell binding assay

The $^{125}$I-labelled-HML-IT-Fab (OST7), $^{125}$I-labelled-Fab (OST7) and $^{125}$I-labelled-HML-IT-Fab (56C) (3–5 ng per 100 µl) were incubated with increasing concentrations of KT005 cells ($2 \times 10^5$–$5 \times 10^6$ per 100 µl) in 5.7 × 46 mm microcentrifuge tubes for 1 h at 4°C. After centrifugation at 10 000 ***g***, the supernatant was aspirated and the tubes were cut. Then the radioactivity bound to the cells was counted in an auto-well gamma counter. Specific binding to the cells was calculated by subtracting the nonspecific binding of $^{125}$I-labelled-HML-IT-Fab (56C) from the binding of $^{125}$I-labelled-HML-IT-Fab (OST7) and $^{125}$I-labelled-Fab (OST7). The binding of 56C to OST7 was less than 3% of the added radioactivity. The immunoreactive fraction of the radiolabelled antibodies was determined by the method described by Lindmo et al (1984).

### Biodistribution studies

Biodistribution studies were performed when the tumours grew to be about 200 mg in weight. Following a report by Kinuya et al (1996*a*), micro-osmotic pumps (Altzet model 1003D, Alza, Palo Alto, CA, USA) filled with 1.2 mg $ml^{-1}$ of [$Asn^{-1}$,$Val^5$]-angiotensin II (Sigma, St. Louis, MO, USA) dissolved in saline were implanted between scapulae of nude mice bearing KT005 xenografts, 4 h before the injection of radiolabelled HML-Fab. Saline was filled in pumps for the control animals. The pumps infused solution at a constant flow rate of 1.0 µl $h^{-1}$ for 72 h under physiological conditions at 37°C. At 1, 3 and 24 h after the administration of antibody, the mice were killed, and their organs were removed, weighed and counted for radioactivity. The micro-osmotic pumps were removed 1 h after the injection of the antibody. As a control study, a fragment of 56C antibody, and for another group, Fab fragment of OST7 radioiodinated by chloramine-T method, were injected with the angiotensin II treatment, and biodistribution was studied at 3 h after the injection. The experimental protocol is shown in Table 1. Data were expressed as percentage of injected dose per gram of tissue, normalized to 20 g mice, and also as tumour-to-normal tissue ratios.

### Immunoscintigraphy

For the imaging of tumour-bearing nude mice, HML was radioiodinated with Na[$^{131}$I], and subsequent conjugation with Fab fragment of OST7 was performed by procedures similar to those used for $^{125}$I-labelled-HML-IT-Fab (OST7). The specific activity of the $^{131}$I-labelled-HML-IT-Fab (OST7) was about 38.2 MBq $mg^{-1}$. As shown in Table 1, after treatment with angiotensin II, 7.7 MBq of $^{131}$I-labelled-HML-IT-Fab (OST7) was administered intravenously via the tail vein. At 3 h after injection of radiolabelled Fab, mice were anaesthetized by intraperitoneal injection of sodium pentobarbital, and scintigrams were obtained using a gamma camera equipped with a pinhole collimator (Hnatowich et al, 1987; Sakahara et al, 1993).

**Table 1** Experimental protocol

| Time | Activity |
|---|---|
| –4 h | Administration of angiotensin II or saline with micro-osmotic pump |
| 0 h | Radioiodinated Fab i.v. |
| 1 h | Removal of micro-osmotic pump |
| 1 h, 3 h or 24 h | Sacrifice and radioactivity count |

© Cancer Research Campaign 1999 

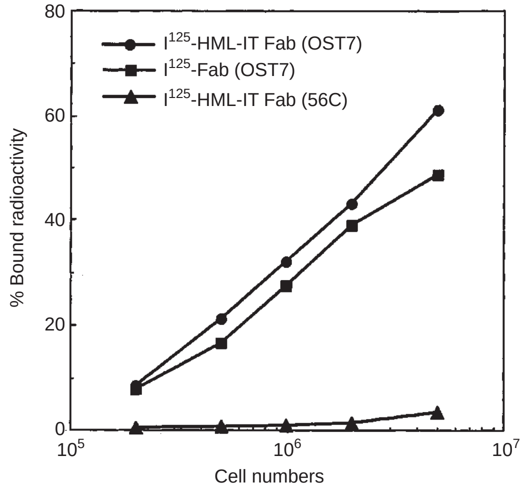

**Figure 1** Binding of $^{125}$I-labelled-HML-IT-Fab (OST7) (●), $^{125}$I-labelled-Fab (OST7) (■) and $^{125}$I-labelled-HML-IT-Fab (56C) (▲) to KT005 cells. The percentage of bound radioactivity is plotted against the number of cells

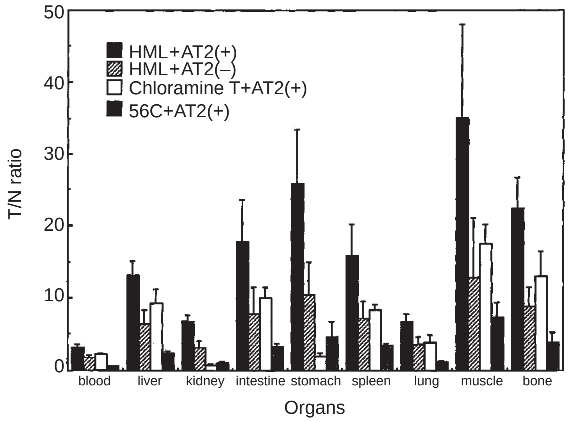

**Figure 2** Tumour-to-normal tissue ratios at 3 h after the injection of radiolabel. When HML method was used as a radioiodination and combined with angiotensin II treatment, we obtained the highest tumour-to-nontumour ratios in each organ

### Statistical analysis

Results were statistically analysed using unpaired t-test for in vivo studies. Differences were considered significant when the $P$ value was less than 0.05.

All animal experiments were carried out in accordance with the Japanese regulations regarding animal care and handling.

### RESULTS

### Cell binding assay

The HML method did not affect the immunoreactivity of OST7 fragment, and there was no significant difference in binding to KT005 cells between $^{125}$I-labelled-HML-IT-Fab (OST7) and $^{125}$I-labelled-Fab (OST7) (see Figure 1). The immunoreactive fractions of the radiolabelled fragments were 67.5% and 61.7%, respectively.

### Biodistribution studies

The biodistribution data of $^{125}$I-labelled-HML-IT-Fab (OST7, 56C) and $^{125}$I-labelled-Fab (OST7) with or without angiotensin II treatment are summarized in Table 2. In addition, Figure 2 shows tumour-to-normal tissue ratios at 3 h after the injection of these radiolabelled fragments. The percentage of injected dose per gram of tissue (%ID $g^{-1}$) of tumours at 1, 3 and 24 h with and without angiotensin treatment after injection of $^{125}$I-labelled-HML-IT-Fab (OST7) were 15.5% and 15.7%, 16.3% and 9.0%, and 3.4% and 3.1%, respectively, and a significant difference in tumour uptake was observed between tumours with and without angiotensin treatment at 3 h after the injection ($P < 0.01$). The %ID $g^{-1}$ in blood and kidneys at 3 h after the injection were 5.1% and 5.3%, 2.5% and 3.0%, respectively. The %ID $g^{-1}$ in blood and kidneys at 3 h of $^{125}$I-labelled-HML-IT-Fab (OST7) labelled by chloramine-T method were 4.2% and 11.5%, respectively, the latter being significantly higher than that of $^{125}$I-labelled-HML-IT-Fab (OST7). The non-specific accumulation of control antibody 56C to the tumour was low at 3 h. High tumour-to-normal tissue ratios ranging from 3.2 (blood) to 35.1 (muscle) were obtained using HML with angiotensin treatment.

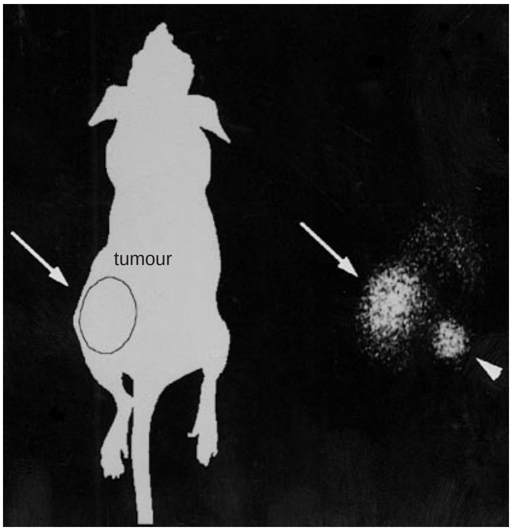

**Figure 3** Scintigram of mouse bearing KT005 human osteogenic sarcoma (pharmacoimmunoscintigraphy). Image was obtained at 3 h after the injection of $^{131}$I-HML-IT-Fab. Tumour (arrow) xenografted at the left back is clearly identified, and normal organs, including the kidney, were not visualized except the urinary bladder (arrowhead)

### Immunoscintigraphy

The scintigraphic images were consistent with the results of biodistribution data (Figure 3). A clear tumour image with low background activity, including kidneys, was obtained as early as 3 h after injection of $^{131}$I-labelled-HML-IT-Fab (OST7).

### DISCUSSION

Immunoscintigraphy and radioimmunotherapy using monoclonal antibody have been used for the detection and treatment of malignant tumours. However, delays caused by slow clearance of unbound antibody from normal tissue and from blood is a problem for both diagnostic and therapeutic applications. Multi-step tumour targeting using a high-affinity binding system such as (strept)avidin-biotin ($10^{15}$ $M^{-1}$) is an efficient method of overcoming some of the problems in tumour targeting. In this approach, a lower molecular weight ligand can be used as a carrier

 © Cancer Research Campaign 1999

**Table 2** Biodistribution data for Fab in KT005-bearing nude mice

| **Antibody (Fab)** | **OST7** | | | | | | **56C** | **OST7** | |
|---|---|---|---|---|---|---|---|---|---|
| **iodination** | **HML** | | | | | | **HML** | **chloramine T** | |
| **time** | **1 h** | | **3 h** | | **24 h** | | **3 h** | **3 h** | |
| **angiotensin II** | **+** | **–** | **+** | **–** | **+** | **–** | **+** | **+** | **–** |
| **group** | **A** | **B** | **C** | **D** | **E** | **F** | **G** | **H** | **I** |
| Blood | 12.73 ± 1.60 | 12.76 ± 2.00 | 5.08 ± 0.46 | 5.29 ± 0.79 | 0.30 ± 0.06 | 0.28 ± 0.03 | 7.93 ± 1.00 | *4.23 ± 0.39 | 4.04 ± 0.32 |
| Liver | 2.84 ± 0.26 | 2.92 ± 0.42 | 1.27 ± 0.38 | 1.34 ± 0.36 | 0.10 ± 0.02 | 0.09 ± 0.03 | 1.98 ± 0.45 | 1.03 ± 0.13 | 0.95 ± 0.14 |
| Kidney | 11.51 ± 2.64 | 11.34 ± 2.45 | 2.53 ± 0.30 | 3.01 ± 0.64 | 0.16 ± 0.05 | 0.14 ± 0.01 | 4.37 ± 0.43 | *11.45 ± 2.12 | 12.91 ± 4.73 |
| Intestine | 2.33 ± 0.74 | 2.35 ± 0.77 | 0.94 ± 0.17 | **1.19 ± 0.30 | 0.12 ± 0.04 | 0.08 ± 0.03 | 1.43 ± 0.15 | 0.95 ± 0.19 | 1.00 ± 0.15 |
| Stomach | 1.33 ± 0.26 | 1.41 ± 0.66 | 0.68 ± 0.25 | 0.89 ± 0.22 | 0.08 ± 0.02 | 0.04 ± 0.01 | 1.14 ± 0.41 | *5.24 ± 1.68 | 5.92 ± 3.15 |
| Spleen | 2.47 ± 0.28 | 2.62 ± 0.21 | 1.06 ± 0.21 | **1.39 ± 0.35 | 0.20 ± 0.06 | 0.14 ± 0.04 | 1.35 ± 0.14 | 1.12 ± 0.18 | 1.02 ± 0.14 |
| Lung | 6.47 ± 1.21 | 5.66 ± 0.99 | 2.47 ± 0.27 | 2.57 ± 0.70 | 0.16 ± 0.04 | 0.18 ± 0.02 | 3.76 ± 0.45 | 2.45 ± 0.39 | 1.85 ± 0.31 |
| Muscle | 0.98 ± 0.29 | 1.14 ± 0.57 | 0.51 ± 0.16 | **0.82 ± 0.29 | 0.14 ± 0.11 | 0.14 ± 0.11 | 0.70 ± 0.24 | 0.54 ± 0.05 | 0.57 ± 0.27 |
| Bone | 1.80 ± 0.39 | 1.89 ± 0.47 | 0.75 ± 0.18 | 1.01 ± 0.46 | 0.21 ± 0.14 | 0.13 ± 0.04 | 1.14 ± 0.26 | 0.75 ± 0.22 | 0.71 ± 0.04 |
| Tumour | 15.49 ± 2.94 | 15.68 ± 3.32 | 16.29 ± 3.21 | *9.03 ± 2.97 | 3.35 ± 0.65 | 3.05 ± 0.59 | 4.59 ± 0.45 | *9.33 ± 1.48 | 8.58 ± 1.80 |
| T/Blood | 1.29 ± 0.33 | 1.23 ± 0.34 | 3.18 ± 0.40 | 1.76 ± 0.31 | 10.56 ± 2.47 | 11.98 ± 2.35 | 0.58 ± 0.08 | 2.20 ± 0.21 | 2.11 ± 0.33 |
| T/Kidney | 1.42 ± 0.36 | 1.39 ± 0.37 | 6.67 ± 0.89 | 3.06 ± 0.98 | 19.76 ± 4.58 | 24.42 ± 4.89 | 1.05 ± 0.07 | 0.82 ± 0.10 | 0.78 ± 0.30 |

Mean ± SD of % ID $g^{-1}$ and its tumour-to-blood or tumour-to-kidney ratio for 4–8 mice; *$P$ < 0.01 compared with group C; **$P$ < 0.05 compared with group C

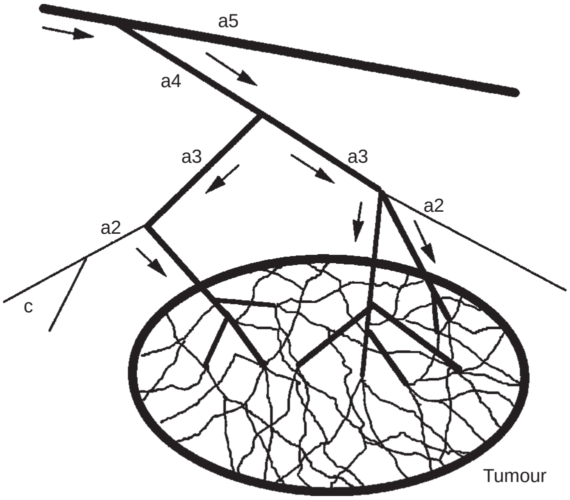

**Figure 4** Schematic representation of the arrangement of arterioles and tumour vessels. Arterioles were classified numerically according to Strahler's nomenclature: c, true capillaries; a2, terminal arterioles; a3–a5, arterioles; arrows, direction of flow of radioisotope for the last step to give higher tumour-to-normal organ ratios sooner, using the rapid clearance of unbound radio-labelled ligand. Some encouraging results have been reported in experimental and clinical studies (Hnatowich et al, 1987; Kalofonos et al, 1990; Paganelli et al, 1991; Saga et al, 1994; Magnani et al, 1996). We also reported an avidin chase method to facilitate the clearance of unbound radiolabelled biotinylated anti-body from the circulation (Kobayashi et al, 1994; Yao et al, 1995). However, these methods require multiple injections, which can be troublesome. Moreover, they can cause the problem of creating a human anti-streptavidin or anti-avidin antibody, as well as human anti-mouse monoclonal antibodies.

Another approach to obtain high tumour-to-background ratios soon after the injection of radiolabelled reagent is the use of fragments, such as Fab and $F(ab')_2$, or single-chain Fv (scFv) as a carrier of radionuclide. Fab and $F(ab')_2$ obtained by enzymatic digestion of immunoglobulins demonstrate more rapid tumour targeting, better penetration, and faster blood clearance than their intact IgG molecules. Also, the absence of an Fc region and short residence in the circulation contribute to lower immunogenicity of antibody fragments in vivo. However, fragments have the disadvantage of high kidney uptake and lower absolute uptake in the tumour than intact IgG (Yokota et al, 1993). Genetically engineered scFv demonstrates much faster clearance from the circulation than Fab and its tumour uptake is very low (Hu et al, 1996), although dimer forms of scFv can increase absolute tumour uptake. In the present study, we used Fab fragment that has an intermediate character between IgG and scFv, obtained the results of increased absolute uptake by angiotensin II treatment, and decreased renal accumulation of radioactivity by a new conjugation technique. These methods could be used for scFv or other antigen-binding small proteins as well as Fab fragments.

Figure 4 schematically illustrates the origin of tumour vasculature from the preexisting vascular bed. Vessel segments are ordered according to Strahler's nomenclature (Strahler et al, 1957). Tumour neovasculature starts from the junction between a3 and a2, and goes on to feed the growing tumour (Hori et al, 1990; Hori et al, 1993). Angiotensin II selectively increases the

© Cancer Research Campaign 1999

resistance of a2 vessels and induces hypertension. Since angiotensin II does not affect the resistance of a3, tumour vasculature originating from the terminal portion of a3 is not affected. Angiotensin II treatment, therefore, selectively redistributes the blood flow to the tumour vascular bed, and it has already been used clinically for induced hypertension chemotherapy. This method can also be applied in antibody-based tumour targeting, and Kinuya et al (1996*a*) reported high tumour accumulation of radiolabelled IgG in mice. We speculated that in induced hypertension, the increase in tumour accumulation would be higher using Fab fragments than intact IgGs due to their smaller molecular size. Indeed, in our study with Fab fragment, HML-Fab conjugate was highly accumulated in the tumour.

HML is a new linker, which was designed to be metabolized in the liver into the *m*-iodohippuric acid and excreted rapidly through the kidneys. In vitro studies showed that HML-protein conjugates were very stable in serum (Wakisaka et al, 1997). The binding affinity of the $^{125}$I-labelled-HML-IT-Fab ($^{125}$I-HML-Fab) to the tumour cells was the same as $^{125}$I-labelled-Fab labelled by chloramin-T method ($^{125}$I-CT-Fab), but the in vivo studies showed that, at 3 h after the injection of radioiodinated Fab with angiotensin II pretreatment, %ID $g^{-1}$ of tumour in $^{125}$I-HML-Fab was significantly higher than that of $^{125}$I-CT-Fab, and %ID $g^{-1}$ in kidneys using $^{125}$I-HML-Fab was significantly lower than that of $^{125}$I-CT-Fab, resulting in a markedly higher tumour-to-kidney ratio using $^{125}$I-HML-Fab. The kidney uptake of $^{125}$I-CT-Fab without angiotensin treatment at 1 h and 24 h were 69.03% and 0.42% (data not shown), respectively. The difference of the radioactivity in the kidneys between $^{125}$I-CT-Fab and $^{125}$I-HML-Fab resulted from clearance of radioiodine shortly after injection. Consequently, the combination of angiotensin II pretreatment and HML-radioiodination method made it possible to get high tumour uptake with low background radioactivity including the kidney, and also, this enables clear tumour imaging soon after injection of radiolabelled Fab fragment.

Although the micro-osmotic pump filled with angiotensin was removed at 1 h after injection of radioiodinated Fab, there was no significant difference between the accumulation of tumour with and without angiotensin treatment at 1 h, and it was not until 3 h after the administration that we recognized the difference between the groups with and without angiotensin treatment. Kinuya et al reported that the increase of tumour blood flow and blood volume were observed just 10 min after the administration of angiotensin II by $^{201}$TI and $^{99m}$Tc-HSA, and the effect of induced hypertension was confirmed to disappear after the removal of the pump filled with angiotensin II (Kinuya et al, 1996*b*; 1997). Therefore, although the exact reason remains unknown, there might be some secondary changes after induced hypertension by angiotensin II that enhance tumour uptake of radiolabelled antibody even after normalization of blood pressure. The uptake of $^{125}$I-HML-Fab to the tumour (16.3%) was significantly higher than that of $^{125}$I-CT-Fab (9.3%) ($P < 0.01$). A reason for this difference may be the increased stability of $^{125}$I-HML-Fab, since $^{125}$I-CT-Fab seemed to be dehalogenated at the tumour site, and free iodine accumulated in the stomach. In addition, there was no statistically significant difference between tumour accumulations of $^{125}$I-CT-Fab with and without angiotensin treatment at 3 h. This may also be due to the instability of $^{125}$I-CT-Fab compared to $^{125}$I-HML-Fab.

Positron emission tomography (PET) has several advantages over conventional scintigraphy, including better spatial resolution and accurate quantification of tissue uptake. Positron emitter halogens, iodine-124 ($^{124}$I) and fluorine-18 ($^{18}$F) might be used for radiolabelling of HML as well as iodine-131. Especially, $^{18}$F is a widely used positron emitter. There have been some reports describing monoclonal antibodies labelled with $^{18}$F (Garg et al, 1992; Vaidyanathan G et al, 1992). According to these reports, tumour-to-normal tissue ratios were not high within a few hours of administration. Therefore, a new conjugate of $^{18}$F-HML-Fab would be more appropriate for PET imaging.

In conclusion, the combined use of a novel metabolizable linker, HML, for radioiodination of Fab, and angiotensin II treatment, demonstrated high accumulation of Fab in the tumour with low background radioactivity, including the kidney, soon after the injection of radiolabel, resulting in successful tumour imaging, which we named pharmacoimmunoscintigraphy. These results suggest that PET scanning using $^{18}$F-labelled-HML-antibody fragment conjugates would be feasible with this technique.

### REFERENCES

Arano Y, Inoue T, Mukai T, Wakisaka K, Sakahara H, Konishi J and Yokoyama A (1994) Discriminated release of a hippurate-like radiometal chelate in nontarget tissues for target-selective radioactivity localization using pH-dependent dissociation of reduced antibody. *J Nucl Med* **35**: 326–333

Collier BD, Abdel Nabi H, Doerr RJ, Harwood SJ, Olsen J, Kaplan EH, Winzelberg GG, Grossman SJ, Krag DN and Mitchell EP (1992) Immunoscintigraphy performed with In-111-labeled CYT-103 in the management of colorectal cancer: comparison with CT. *Radiology* **185**: 179–186

Corbisiero RM, Yamauchi DM, Williams LE, Esteban JM, Odom Maryon T and Beatty JD (1991) Comparison of immunoscintigraphy and computerized tomography in identifying colorectal cancer: individual lesion analysis. *Cancer Res* **51**: 5704–5711

Garg PK, Garg S, Bigner DD and Zalutsky MR (1992) Localization of fluorine-18-labeled Mel-14 monoclonal antibody $F(ab')_2$ fragment in a subcutaneous xenograft model. *Cancer Res* **52**: 5054–5060

Gasparini M, Buraggi GL, Regalia E, Maffioli L, Balzarini L and Gennari L (1994) Comparison of radioimmunodetection with other imaging methods in evaluating local relapses of colorectal carcinoma. *Cancer* **73**: 846–849

Greenwood FC, Hunter WN and Glover JS (1963) The preparation of $^{131}$I-labeled human growth hormone of high specific radioactivity. *Biochemistry* **89**: 114–123

Hnatowich DJ, Virzi F and Rusckowski M (1987) Investigation of avidin and biotin for imaging applications. *J Nucl Med* **28**: 1294–1302

Hori K, Suzuki M, Abe I, Saito S and Sato H (1985) Increase in tumor vascular area due to increased blood flow by angiotensin II in rats. *J Natl Cancer Inst* **74**: 453–459

Hori K, Suzuki M, Tanda S and Saito S (1990) In vivo analysis of tumor vascularization in the rat. *Jpn J Cancer Res* **81**: 279–288

Hori K, Zhang QH, Saito S, Tanda S, Li HC and Suzuki M (1993) Microvascular mechanisms of change in tumor blood flow due to angiotensin II, epinephrine, and methoxamine: a functional morphometric study. *Cancer Res* **53**: 5528–5534

Hosoi S, Nakamura T, Higashi S, Yamamuro T, Toyama S, Shinomiya K and Mikawa H (1982) Detection of human osteosarcoma-associated antigen(s) by monoclonal antibodies. *Cancer Res* **42**: 654–659

Hu SZ, Shively L, Raubitschek A, Sherman M, Williams LE, Wong JYC, Shively JE and Wu AM (1996) Minibody: a novel engineered anti-carcinoembryonic antigen antibody fragment (single-chain Fv-$C_H$3) which exhibits rapid, high-level targeting of xenografts. *Cancer Res* **56**: 3055–3061

Hunter WN and Greenwood FC (1962) Preparation of iodine-131 labeled human growth hormone of high specific activity. *Nature* **164**: 495–496

Kalofonos HP, Rusckowski M, Siebecker DA, Sivolapenko GB, Snook D, Lavender JP, Epenetos AA and Hnatowich DJ (1990) Imaging of tumor in patients with indium-111-labeled biotin and streptavidin-conjugated antibodies: preliminary communication. *J Nucl Med* **31**: 1791–1796

Kinuya S, Yokoyama K, Konishi S, Tonami N and Hisada K (1996*a*) Effect of induced hypertension with angiotensin II infusion on biodistribution of $^{111}$In-labeled monoclonal antibody. *Nucl Med Biol* **23**: 137–140

Kinuya S, Yokoyama K, Konishi S, Hwang EH, Takayama T, Michigishi T and Tonami N (1996*b*) $^{201}$Tl and $^{99m}$Tc-human serum albumin for assessment of the

 *© Cancer Research Campaign 1999*

effect of hypertensive treatment with angiotensin II infusion on tumour circulation. *Nucl Med Commun* **17**: 160–163

Kinuya S, Yokoyama K, Yamamoto W, Konishi S, Shuke N, Aburano T, Watanabe N, Takayama T, Michigishi T and Tonami N (1997) Short-period-induced hypertension could improve tumor-to-nontumor ratios of radiolabeled monoclonal antibody. *Nucl Med Biol* **24**: 547–551

Kobayashi H, Sakahara H, Hosono M, Shirato M, Konishi J, Takahashi JA, Oda Y, Kikuchi H, Endo K, Kozai Y and Hatanaka M (1993) Scintigraphic detection of xenografted tumors producing human basic fibroblast growth factor. *Cancer Immunol Immunother* **37**: 281–285

Kobayashi H, Sakahara H, Hosono M, Yao ZS, Toyama S, Endo K and Konishi J (1994) Improved clearance of radiolabeled biotinylated monoclonal antibody following the infusion of avidin as a “chase” without decreased accumulation in the target tumor. *J Nucl Med* **35**: 1677–1684

Lindmo T, Boven E, Cuttitta F, Fedorko J and Bunn PA Jr (1984) Determination of the immunoreactive fraction of radiolabeled monoclonal antibodies by linear extrapolation to binding at infinite antigen excess. *J Immunol Methods* **72**: 77–89

Magnani P, Paganelli G, Modorati G, Zito F, Songini C, Sudati F, Koch P, Maecke HR, Brancato R, Siccardi AG and Fazio F (1996) Quantitative comparison of direct antibody labeling and tumor pretargeting in uveal melanoma. *J Nucl Med* **37**: 967–971

Meares CF, McCall MJ, Reardan DT, Goodwin DA, Diamanti CI and McTigue M (1984) Conjugation of antibodies with bifunctional chelating agents: isothiocyanate and bromoacetamide reagents, methods of analysis, and subsequent addition of metal ions. *Anal Biochem* **142**: 68–78

Nakamura T, Gross M, Yamamuro T and Liao SK (1987) Identification of a human osteosarcoma-associated glycoprotein with monoclonal antibodies: relationship with alkaline phosphatase. *Biochem Cell Biol* **65**: 1091–1100

Paganelli G, Magnani P, Zito F, Villa E, Sudati F, Lopalco L, Rossetti C, Malcovati M, Chiolerio F, Seccamani E, Siccardi AG and Fazio F (1991) Three-step monoclonal antibody tumor targeting in carcinoembryonic antigen-positive patients. *Cancer Res* **51**: 5960–5966

Saga T, Weinstein JN, Jeong JM, Heya T, Lee JT, Le N, Paik CH, Sung C and Neumann RD (1994) Two-step targeting of experimental lung metastases with biotinylated antibody and radiolabeled streptavidin. *Cancer Res* **54**: 2160–2165

Sakahara H, Endo K, Nakashima T, Koizumi M, Kunimatsu M, Kawamura Y, Ohta H, Nakamura T, Tanaka H, Kotoura Y, Yamamuro T, Hosoi S, Toyama S and Torizuka K (1987) Localization of human osteogenic sarcoma xenografts in nude mice by a monoclonal antibody labeled with radioiodine and indium-111. *J Nucl Med* **28**: 342–348

Sakahara H, Saga T, Endo K, Hattori N, Hosono M, Kobayashi H, Shirato M, Yamamuro T, Toyama S, Arano Y, Yokoyama A and Konishi J (1993) In vivo instability of reduction-mediated 99mTc-labeled monoclonal antibody. *Nucl Med Biol* **20**: 617–623

Sato H, Sugiyama K, Hoshi M, Urushiyama M and Ishizuka K (1995) Angiotensin II (AII) induced hypertension chemotherapy (IHC) for unresectable gastric cancer: with reference to resection after down staging. *World J Surg* **19**: 836–842

Strahler AN (1957) Quantitative analysis of watershed geomorphology. *Trans Am Geophys Union* **38**: 913–920

Suzuki M, Hori K, Abe I, Saito S and Sato H (1981) A new approach to cancer chemotherapy: selective enhancement of tumor blood flow with angiotensin II. *J Natl Cancer Inst* **67**: 663–669

Tanaka C, Yamamuro T, Masuda T, Tanaka H, Matsumoto M, Kotoura Y, Tsuji T, Toguchida J, Miyama Inaba M and Ueda M (1986) Recognition of serum alkaline phosphatase by murine monoclonal antibodies against human osteosarcoma cells. *Cancer Res* **46**: 4853–4857

Vaidyanathan G, Bigner DD and Zalutsky MR (1992) Fluorine-18-labeled monoclonal antibody fragments: a potential approach for combining radioimmunoscintigraphy and positron emission tomography. *J Nucl Med* **33**: 1535–1541

Wakisaka K, Arano Y, Uezono T, Akizawa H, Ono M, Kawai K, Ohomomo Y, Nakayama M and Saji H (1997) A novel radioiodination reagent for protein radiopharmaceuticals with L-lysine as a plasma-stable metabolizable linkage to liberate m-iodohippuric acid after lysosomal proteolysis. *J Med Chem* **40**: 2643–2652

Yao Z, Zhang M, Kobayashi H, Sakahara H, Nakada H, Yamashina I and Konishi J (1995) Improved targeting of radiolabeled streptavidin in tumors pretargeted with biotinylated monoclonal antibodies through an avidin chase. *J Nucl Med* **36**: 837–841

Yokota T, Milenic DE, Whitlow M, Wood JF, Hubert SL and Schlom J (1993) Microautoradiographic analysis of the normal organ distribution of radioiodinated single-chain Fv and other immunoglobulin forms. *Cancer Res* **53**: 3776–3783

*© Cancer Research Campaign 1999*
